# Dimensional models of personality disorders: Challenges and opportunities

**DOI:** 10.3389/fpsyt.2023.1098452

**Published:** 2023-03-07

**Authors:** Conal Monaghan, Boris Bizumic

**Affiliations:** Research School of Psychology, Australian National University, Canberra, ACT, Australia

**Keywords:** personality disorder, dimensional, psychometrics, clinical utility, cross-cultural, stigma, severity, traits

## Abstract

Categorical models of personality disorders have been beneficial throughout psychiatric history, providing a mechanism for organizing and communicating research and treatment. However, the view that individuals with personality disorders are qualitatively distinct from the general population is no longer tenable. This perspective has amassed steady criticism, ranging from inconsequential to irreconcilable. In response, stronger evidence has been accumulated in support of a dimensional perspective that unifies normal and pathological personality on underlying trait continua. Contemporary nosology has largely shifted toward this dimensional perspective, yet broader adoption within public lexicon and routine clinical practice appears slow. This review focuses on challenges and the related opportunities of moving toward dimensional models in personality disorder research and practice. First, we highlight the need for ongoing development of a broader array of measurement methods, ideally facilitating multimethod assessments that reduce biases associated with any single methodology. These efforts should also include measurement across both poles of each trait, intensive longitudinal studies, and more deeply considering social desirability. Second, wider communication and training in dimensional approaches is needed for individuals working in mental health. This will require clear demonstrations of incremental treatment efficacy and structured public health rebates. Third, we should embrace cultural and geographic diversity, and investigate how unifying humanity may reduce the stigma and shame currently generated by arbitrarily labeling an individual's personality as normal or abnormal. This review aims to organize ongoing research efforts toward broader and routine usage of dimensional perspectives within research and clinical spaces.

## 1. Introduction

Classical views of personality disorder (PD) as discrete categories have played an important role in understanding and communicating psychopathology throughout history. The benefits of this perspective are enticing: a contained organization of symptoms to facilitate standardized research, organize public awareness and stigma reduction campaigns, allocate public health funding and appropriate treatment intensities, and normalize clear labels for communicating patient formulations (a description of symptoms and their inter-relationships) to professionals and families. It is no wonder this nosology was retained in the Diagnostic and Statistical Manual of Mental Disorders 5th Edition [DSM-5; (1)].

The accurate diagnosis and classification of PD is vital to developing a strong health-care system. PD is considered chronic and relatively resistant to current treatments, with a large proportion of individuals still retaining their disorder after extended periods of treatment [e.g., (2–4)]. Consequently, PD has a relatively poor prognosis (depending on kind and severity) and reduces treatment efficacy of any co-morbid mental health issues (5). This pervasiveness places a substantial burden on the time and finances of already stretched health-care systems (6–9). PD's impact at both the individual and community level necessitates a diagnostic system that is grounded in strong evidence-based research and that facilitates effective treatment approaches, regardless of the allure of familiarity or maintaining the *status quo*.

Since its inception, the categorical system has steadily accumulated criticism (10, 11), ranging from inconsequential to irreconcilable. Considerable attempts have been unable to reproduce the factor structure of the DSM-IV-TR's categorical model (12). The absence of a stable factor structure suggests that the categorical structure cannot robustly describe the architecture of personality psychopathology. Issues with factorial replication are exacerbated by the substantial symptom overlap between disorders that facilitates their excessive and unwarranted co-occurrence (13). As a result, individuals are substantially more likely to be diagnosed with several PDs than a singular one (14), weakening the argument that categories provide neat constellations of inter-related symptoms. Equally, this approach appears unable to accurately capture the full range of personality psychopathology. Estimates of patients who do not fit neatly into current categories range from 21 to 49%, accordingly given the general diagnosis of Personality Disorder – Not Otherwise Specified (PD-NOS) (15). PD-NOS also appears to be in regular usage to describe mixed or complex presentations given the difficulties in classifying individuals within the current framework.

Setting standardized diagnostic thresholds (based upon polythetic symptoms) is difficult particularly when each symptom is given equal weighting. This means that individuals with the same number of symptoms can have substantially different levels of distress. Between each PD, diagnostic thresholds occur at different levels of pathology [latent trait locations; (16)], suggesting a need for further standardization. Due to these issues, it is likely that many clinicians use their clinical judgment based upon an internalized representation of the disorder when making diagnoses. Although the careful application of clinical judgment is vital to making well-informed diagnoses, judgment alone lends itself to bias and inconsistency when not grounded in evidence-based actuarial assessment (17). Taken as a whole, the current categorical approach falls short of fully representing personality psychopathology and providing a scientifically robust understanding of what personality is and what disorders of personality are.

In this review, we discuss challenges and related opportunities of a contemporary and evidence-based PD classification system that addresses many of limitations of the categorical approach, the dimensional model. We wish to acknowledge other reviews regarding additional challenges and barriers including the future of severity and impairment measurement [see (18)], the location and stability of Anankastic and Psychoticism and the interstatiality of lower-order facets (19, 20), utility of hybrid approaches that combine traits indicative of personality disorder prototypes (21, 22), resolving taxometric issues (23), and broader discussions surrounding clinical utility and treatment frameworks (24). Here, we briefly outline the current landscape in terms of shifting from a categorical to dimensional model before highlighting the current measurement issues and suggested advancements, increasing clinician awareness and integration into health-care systems, cross-cultural development, and the potential for stigma reduction (which are summarized in Table 1). We hope this will help to organize research efforts to advance the transition into a robust dimensional framework.

**Table 1:** Summary of challenges and opportunities for research directions.

**Domain**	**Challenges**	**Opportunities for research directions**
Measurement	Expanding measurement approaches	Specifically focus on multimodal approaches in both research and practice. Integrating findings using multitrait-multimethod matrices where possible. Consider how results are informed and limited by chosen methodology directly within test interpretation. Continue to develop joint severity and trait measures to answer pressing research questions around their inter-relationship, utility, and structure.
	Bipolar measurement	Control for social desirability through scales with balanced wording. Increase research into bipolar perspectives to ensure full trait coverage. Further investigate whether maladaptive traits are extremes of normal traits, or normal traits plus dysfunction. Ongoing research should also consider severity/impairment as bipolar.
	Efficiency	Continue to investigate the viability of more efficient measurement tools and approaches such as Computerized Adaptive Tests.
Clinical utility	Treatment development	Organize current evidence-based treatments around trait and interpersonal disorder models. This involves mapping proven interventions to specific traits, facets, and interpersonal issues.
	Training	Integrate dimensional approaches into graduate training programs to increase familiarity and usage, which will also provide naturalistic clinical utility data.
	Treatment evidence	Specific field-trials of whether dimensional approaches outperform categorical approaches for treatment outcomes.
	Health system integration	Directly establish health care support and prognosis related to PD severity for health care system funding.
Inclusivity	Universality	Develop cross-cultural models of PD instead of models imposed from Western contexts onto others.
	Inclusion and stigma	Further research stigma reduction through dimensional perspectives. Consider the benefits of *interpersonal disorders* over *personality disorder*.

## 2. The current landscape of dimensional approaches to personality disorders

The view that individuals with personality disorders (PD) are qualitatively distinct from the general population is no longer tenable. In search for alternative approaches to this diagnostic puzzle, support has amassed for dimensional frameworks, which suggest that humans differ in degree not in kind. Within this perspective, PD occurs at maladaptive extremes of the standard personality traits all humans share (25, 26) and as specific combinations of these trait extremes. The degree of life impairment forms the basis for a PD diagnosis. This approach has gained substantial support by much more than a vocal minority, with broad calls and movements toward mainstream adoption [see (12, 27, 28)].

Despite some important differences in the prevailing approaches, dimensional models of PD typically consider two key criteria: *severity* and *style*. Severity captures the core distress that is common to all PDs, its impact on the individual's self-direction and identity (intrapersonal functioning), as well as their ability to form close relationships and empathize with others (interpersonal functioning). Indices of global severity are robust predictors of both the presence of a personality disorder and prognosis, and track with fluctuations in clinical functioning [e.g., (29–32)]. According to the International Classification of Diseases' eleventh revision [ICD-11; (33)], severity is the key and sole requirement for making a diagnosis of PD (34, 35). The central placement of impairment is grounded in research that global severity ratings are sensitive and specific predictors of PD, and provide better estimates of clinician-rated psychosocial impairment than specific categorical diagnoses do (36, 37). It appears that the severity of personality disorder (i.e., mild, moderate, severe) is more indicative of dysfunction and outcomes than the specific typology of the disorder.

The second criterion describes the stylistic features of the presentation, largely in relation to some derivation of the Five-Factor Model (FFM) of personality (38, 39). Although the DSM-5 officially retained a categorical approach, the DSM-5's Alternative Model of Personality Disorders (AMPD) Criterion B comprises the traits of negative affectivity (continua from emotional stability to neuroticism), detachment (introversion to extroversion), antagonism (agreeableness to antagonism), disinhibition (conscientiousness to impulsivity), and psychoticism (closed to experience to open to experience). The DSM-5's approach to diagnosing PD in the AMPD differs from the ICD-11 as it requires the presence of one or more elevated traits. Nevertheless, there is a growing interest in using only Criterion A for understanding, diagnosing, and managing PD [see (18)]. FFM has historically demonstrated a good resilience to criticism, providing meaningful inferences about individual difference grounded in hereditable genetic underpinnings (40) aligned with biological systems (41). The five basic traits have rank-order stability across time (42) and are relatively reproducible cross-culturally [(43); but see (44–46) which question the universality of the model]. The FFM provides an excellent candidate for explaining all personality variation, with current dimensional PD models capturing dysfunctional versions or extremes of these traits (47).

In the present paper we particularly focus on the dimensional frameworks grounded in the FFM given their current prominence in diagnostic nosology, and given that the lexical origins of the FFM support its universality at describing population level individual differences. Nonetheless, this does not mean they are the only alternative to categorical perspectives, or that they necessarily capture underlying biological, neurological, and neurochemical systems. Evolutionary and neurological / neurochemical processes can be mapped onto FFM traits [see (41)] yet the FFM has weaker support for capturing processes *within* people than it does *between* them (48). The Function Ensemble of Temperaments [FET; (49)] emphasizes the importance of distinguishing temperament, the neurobiological processes that underly behavioral and emotional regulation, from personality, the socio-cultural integration of attitudes, values, and personal experience. Evolutionary pressures shape the functional and dynamic neuroanatomic and neurochemical systems that drive temperament, and psychopathology arises from the failure of these systems to meet specific situational demands. Although this is similar to FFM based person-environment transactional models [e.g., (50)], FET's neurological systems do not map neatly onto the FFM, and its proponents argue that the high interconnectivity between emotional and energetic regulatory systems are more complex than DSM/ ICD taxonomies suggest (51). We, therefore, need ongoing efforts to identify a coherence between FET neurobiology behavior and dimensional manifestations of personality.

Within the FFM taxonomy, evidence for the dimensional approach is largely focused on the Criterion B of the AMPD given the time that has passed since its inception (released 2013) when compared to the ICD-11 (33). The structure of the AMPD, which is principally based on research with the Personality Inventory for DSM-5 (PID-5) (52), has demonstrated a stable and reproducible factor structure across studies and populations, with appropriate estimates of internal consistency for content breadth and scale length (19, 53–55). Importantly, dimensional approaches are largely able to reproduce categorical diagnosis (56), provide incremental validity by describing all personality (removing PD-NOS), and contain more useful and detailed information for treatment planning and monitoring of severity and impairment [e.g., (57)].

Categorical approaches continue to be widely used in research and clinical practice. The resistance to adopting dimensional methodology and language is interesting given broad support that PD is best conceptualized as dimensional by researchers [e.g., (28)] and clinicians (58–62). We now turn to three areas of ongoing inquiry that are impeding the transition into dimensionality, which include both the strengthening of existing frameworks and direct tests of their efficacy and viability.

## 3. Measuring personality disorders: Challenges and opportunities

### 3.1. Expanding measurement approaches

Strengthening measurement precision requires increased usage of a broader array of psychometric tools and approaches. To this end, a range of instruments have been developed to measure the DSM-5 AMPD Level of Functioning Scale (LPFS). The LPFS-Self-Report [LPFS-SR; (63)] captures one generalized index of PD severity (64), comprising impairments of adaptive self and interpersonal functioning. Each level of gradation falls on a range from little or no impairment (0) to extreme impairment (4), with moderate impairment (2) demarcating the threshold for the presence of notable PD concerns. Recently, we have also seen LPFS adaptations, including brief forms [e.g., LPFS-BF; (65, 66)], and versions for specific populations [e.g., the Levels of Personality Functioning Questionnaire for adolecents; (67)]. Semi-structured interviews have also been developed, including the Semi-Structured Interview for Personality Functioning for the DSM-5 [STiP-5.1; (68)] and the Structured Interview for the Level of Personality Functioning scale [SCID-AMPD Module I; (69)]. Specific disorder related impairment measures have also been developed, but their substantial shared variance suggests minimal incremental validity over a singular severity index (36).

There is ongoing debate about the structure of severity in AMPD Criterion A. Some researchers suggests that the LPFS can capture a singular underlying severity construct that can be further divided into strongly correlated intrapersonal and interpersonal components (18, 64), whereas others suggest that a substantial revision is required (70). Individuals vary in how their personality style causes impairments in their wellbeing. For one individual, their personality may specifically impact adaptive interpersonal interactions, whereas for another, it may influence both interpersonal and intrapersonal domains. Guidelines may need to be developed for determining when the severity of PD is interpretable (i.e., when the impact on both domains is similar) or invalid (i.e., when the impairment is domain-specific) (71). This is similar to recommendations for determining the validity of a singular index of cognitive ability in many of the dominant instruments.

An associated issue is the conceptual and empirical overlap between style or traits and severity. Although severity should represent the life impairment that is common among all PD and style should describe the specific nuances of that presentation, they have been difficult to distinguish empirically (72). For example, Hopwood et al. (64) found that each of the four major personality traits mapped onto the LPFS components (disinhibition with self-direction, antagonism with empathy, detachment with intimacy, and neuroticism with identity). Beyond the theoretical difficulties of distinguishing an individual's personality from its influence, scale content is not cleanly differentiated. For example, the severity item from the LPFS-SR “Getting close to others has little appeal to me” shares substantial content with trait item “I don't deal with people unless I have to” from the Personality Inventory for the DSM-5 [PID-5; (52)]. Instead of relying on maladaptive traits, alternative approaches include using normal range traits plus severity to understand personality disorders [e.g., (73)], or considering severity as a measure of an individual's capacity to meet the demands of their environment resulting in personality trait expression (18, 71). Ongoing research and theory are needed to reconcile these issues and to better understand how severity and style interweave and complement each other. Longitudinal designs show promising potential for tackling these problems, particularly in capturing dynamic changes over time [e.g., (74)].

Current measurement of personality disorder traits is heavily reliant on the PID-5 (52). Alternative measures have received substantially less attention, such as the informant (75) and structured interview (76). This has created a psychometric environment where the PID-5's conceptualization and psychometric properties dominate our understanding of dimensional personality, particularly the AMPD (19). The reliance is not surprising given the PID-5 was released with the DSM-5 for AMPD assessment and has substantive time to accrue support for its quite robust psychometric properties (52). Although revisions and refinements to the PID-5 are useful [see (19)], robust measurement requires disconnecting measurement from the construct itself (77). Developing additional measures is vital to overcome weaknesses and biases in a singular approach, regardless of the strength of any measure. As a starting point, a wider variety of self-report instruments should be employed with variations in trait conceptualization (25), such as the Schedule for Nonadaptive and Adaptive Personality [SNAP; (78)] and the Comprehensive Assessment of Traits Relevant to Personality Disorders [CAT-PD; (79)].

Measures are emerging specifically for the ICD-11 PD model, with several being developed from earlier working versions of the ICD-11. These are in addition to the range of measures that were adapted from the PID-5 [e.g., (80, 81)]. Bach et al. (82) developed the PDS-ICD-11 for an updated and psychometrically robust measure of personality severity in line with the published ICD-11 diagnostic criteria. The PDS-ICD-11 captures cognitive, behavioral, and emotional manifestations of self and interpersonal functioning. This scale appears to maximize discrimination in the 0–2.5 range making it an excellent candidate for understanding clinical populations.

The Personality Inventory for the ICD-11 (PiCD-11) was developed from draft trait descriptors (83), and more recent studies have supported the viability of four- and five-factor solutions (with disinhibition and anankastia as bipolar ends of the same trait) and strong convergence between self-rated and clinician-rated solutions (84). A four-factor structure mirrors the AMPD, but with the exclusion of the psychoticism trait. The PiCD-11 has potential to become a dominant ICD-11 severity measure due to its reproducible and robust psychometric properties (85, 86) and consistency between clinician evaluations and self-reported data. Further studies should examine its usefulness and ability to detect meaningful change in larger clinical settings.

Clark et al. (87) developed preliminary scales to capture both dysfunction severity and trait specifiers. This represents the first complete ICD-11 specific measure based on the final clinical diagnostic guidelines and descriptions. The measure combines both severity and traits to promote ongoing research into the differentiation between severity and trait specifiers from both research and clinical perspectives. The researchers stressed the importance of this integration, as it enables a more comprehensive examination of the relationship between severity and traits, and whether they are best understood as one construct or two. Additionally, it allows for the identification and isolation of global biases and confounds, and the ability to continually refine the measure over time, to better understand the relationship between severity and traits. Initial principal axis factoring suggests that both severity and traits can be described by two internalizing-pathology dimensions (Self Dysfunction and Interpersonal Dysfunction) and a single externalizing pathology dimension. We encourage ongoing studies into a collective effort to revise these preliminary scales (currently unnamed to emphasize its ongoing development) with a focus on removing redundancy (where possible) and more advanced analyses (such as confirmatory factor analysis and item response theory).

Mirroring broader psychological research, severity and trait research relies heavily on a mono-method approach using self-report data (88). Bornstein (88) systematically reviewed studies on PD from five major journals from 1991 to 2000, finding that only 8% directly observed behavior, whereas 80% relied exclusively on self-report data. Unfortunately, this issue appears even worse for measures of AMPD Criterion A (severity) than B (traits) (57). This is not surprising given the ease of administration and efficiency of cross-sectional self-report methods. Nevertheless, the construct validity (89) of any personality trait necessitates agreement between observations from a variety of methods. Multi-method approaches are desperately needed because it is currently difficult, if not impossible, to separate trait variance from the measurement properties (error/biases) (90). This issue is exacerbated further by the reliance on a singular instrument (19) and the substantial error and non-trait variance identified in survey methodology [e.g., (91–93)].

In addition to reducing method-specific error, multimethod assessment can yield much richer and more interesting information than a single approach alone could have provided: more than the sum of its parts. For example, diverging trait estimates between two different informants could indicate inconsistent or deceitful behavior, whereas discrepancy between self-report and performance could indicate unrecognized issues or overly critical self-evaluation [e.g., (94)]. Multi-method approaches are rarely used for studies of severity or trait (57), and even when multiple methods are used, they are rarely leveraged to their full potential. Multitrait-multimethod (MTMM) matrices, or contemporary latent modeling equivalents, provide the necessary tools for integrating trait observations from multiple sources to model construct validity. Multiple methods assessing the same trait should converge to provide similar estimates (convergent validity) and the same method assessing distinct constructs should diverge (divergent validity). By integrating correlations between multiple methods and distinct constructs, MTMM can differentiate substantive variance (trait of interest) from methodology-specific variance and random error (90). Unfortunately, correlations between multiple methods are often low to moderate (95, 96) suggesting, at best, we have an incomplete model of personality particularly if we continue to rely on testing validity through convergence between self-reports.

The characteristics of each approach need to be deeply considered when interpreting assessment results. Regrettably, the limitations and biases of the chosen methodology are often an afterthought. Self-reports require participants to be able and willing to introspect honestly or account for actions and experiences. Depending on the trait of interest, informant reports can provide incremental validity to self-report [e.g., (97)] because they can overcome social desirability and recall biases. Nonetheless, interpretation of informant reports should account for an informant's motivations, relationship to the participant, and the rarity of the observed behavior. Standardized interviews can provide rich and nuanced data; however, clinicians are not immune to the biases contaminating self-reports and informant reports [spurring the clinical judgment vs. actuarial debate; (17)]. Whether consciously or unconsciously, clinicians tend to seek confirmatory evidence and to discount contrary evidence in line with their original hypothesis (98). Similar considerations should be taken in interpreting other methodologies, such as life narratives [autobiographies; (99)], direct behavioral observations, and biological or neurological data [see (100), for multimethod clinical assessment]. The strengths and limitations of each approach need to be carefully considered during study design and assessment interpretation, instead of being only referred to as a study limitation.

Important advances have been generated by researchers who moved away from cross-sectional self-report designs. For example, experience sampling has shown that individuals differ in both their trait levels and how much their traits fluctuate daily. The degree of fluctuation is different between people but relatively stable within each individual suggesting differences in both mean trait level and trait reactivity (101). Longitudinal analyses have also demonstrated a demarcation between relatively stable traits and more variable levels of dysfunction. Therefore, while trait levels may remain relatively stable over time, the associated distress is malleable (31). Moving away from cross-sectional mono-method designs will allow researchers to better understand this important, trait vs. severity, issue by separating an individual's traits from their impairment and situational demands (not possible using cross-sectional designs). We encourage future researchers to integrate longitudinal designs and multiple sources of information through MTMM.

An example of the benefits of broader methodological designs and MTMM methodology is the recent clarification of the status of a general factor of psychopathology [“*p*-factor”; (102, 103)], a general tendency to experience persistent psychiatric problems that facilitate life impairment. A common mechanism underlying all psychopathology (e.g., emotional regulation issues or cognitive biases) would be empirically interesting and clinically useful, suggesting a universal target for treatment. Several MTMM studies, however, have suggested that the shared variance between the higher-order internalizing and externalizing factors, and between the lower-level traits, is substantially reduced when accounting for methodology (104, 105). The instability of this factor across measures and samples suggests that this factor is likely the combination of general distress or impairment and a combination of errors associated with that methodology (such as survey-methodology specific error, instrument specific factors, socially desirable responding/halo effect) (31, 92, 106–109). Therefore, MTMM research has substantially weakened the evidence for a general factor of psychopathology and will likely shine light on many current issues in PD research.

A final consideration is an increased emphasis on deriving assessment and nosology based in biological, neurological, and neurochemical observations. Although FFM traits describe individual differences that are important within society, this does not necessarily mean they are grounded in independent biological mechanisms. Multidisciplinary efforts can make important advancements in this domain, particularly through integrating neurology and genetics with advanced computational mathematics. For example, the complexity of highly interconnected regulatory systems likely means that standard linear correlational analyses, including structural equation modeling, may not be sufficient to advance these models further. Instead, we are now at the stage where complex and dynamic non-linear approaches are needed. This might include identifying contingent systems using time series and constructivist approaches [e.g., (110)] to overcome the limitations of current statistical methodology.

### 3.2. Measuring the bipolar nature of personality disorders

Research programs need to further investigate the disparity between the bipolar nature of normal personality and the largely unipolar models of PD. Currently, only one end of each trait continua is associated with distress and dysfunction, whereas the other is considered healthy and resilient. This conceptualization contradicts FFM research and theory on the maladaptively associated with both poles of each trait (47, 111). For example, AMPD antagonism ranges from the adaptive agreeableness pole to a maladaptive antagonistic pole. Generally, it is easier to imagine distress at one pole more than the other. For example, one can more easily perceive distress arising in a person who is highly aggressive, callous, mistrustful, or arrogant than in a person who is highly sympathetic, trusting, benevolent, or modest. Reviews of FFM trait terms have demonstrated that the vast majority of maladaptive words occur only at one pole on each trait (e.g., only 17% of agreeableness terms were maladaptive) (112, 113). Nevertheless, it appears unfeasible that distress does not occur at both ends (114), as someone who is overly trusting and gullible may have difficulties getting their needs met and be vulnerable to exploitation.

The development of the current unipolar perspective is not surprising given earlier [e.g., (52)] and contemporary [e.g., (115)] studies have associated lower quality of life and dysfunction with only the “maladaptive” pole. This has resulted in prevailing measures focusing their measurement accuracy at only one pole of each trait (116–118). Several studies have reworded items to balance social desirability at both poles either by making both poles maladaptive or adaptive. In doing so, these studies have drastically increased associations with categorical disorders located at these “adaptive” poles (particularly Obsessive-Compulsive PD and Psychopathy) and identified dysfunction and impairment with most poles (113, 119, 120). Continued research is needed to identify whether the major domains of personality have only one pathological pole, or whether their bipolarity has been obscured by social desirability and biased item language.

Difficulty modeling bipolar traits is a frequent psychometric issue that is exacerbated because both poles theoretically correlate with general distress [e.g., (121)]. Although the nature of the distress at both poles is likely to differ in intensity and kind, the general distress shared by both poles reduces their distinctiveness, impeding modeling efforts (122). This is a challenge that could be managed through modifications in item wording and by removing distress, or simply estimating bipolar method variance [e.g., (123)], through bifactor measurement models. For example, a marker approach estimates method and error variance (such as social desirability or dysfunction/severity) directly within the model, effectively partialling substantive trait variance from method variance (124). Structural models can then differentiate between the “purer” trait latent factor and external variables of interest, while being cautious not to remove core trait content (125). This might prove difficult if maladaptive traits are simply normal range traits with the addition of personality dysfunction [see (73)].

It is important to differentiate between trait bipolarity and severity bipolarity. The PDS-ICD-11 (84) conceptualizes the emotional and behavioral aspects of the self- and interpersonal severity as bipolar continuums. Opposing poles represent under-controlled and over-controlled aspects of each personality disturbance, with a neutral or middle response reflecting normal functioning. For example, self-worth can range from feeling superior to others to feeling inferior to others. Therefore, dysfunction can occur when there is a mismatch between the individual's adaptive control and the demands of their environment. Subsequent studies using this bipolar scoring scheme have demonstrated substantial advantages of this approach, for example, demonstrating that anankastia is associated with unreachable goals, and that disinhibition reduces the likelihood of individuals reaching planned goals (85, 86). This may represent an opportunity to investigate the intersection between bipolar traits and impairment.

Similarly to bipolar severity, a bipolar trait perspective would also provide clinicians the tools to focus on strengths. PID-5 development team (52) acknowledged that their focus on maladaptive trait ranges came at the cost of measurement precision at more adaptive ranges. Adaptive ranges could act as potential points of stability and strength to counterbalance the vulnerability caused by trait extremes. Curvilinear modeling on bipolar traits will help to identify adaptive trait levels to act as strengths (hyperbolic inflection points), instead of assuming the total absence of the maladaptive pole is a strength. Additionally, curvilinear modeling for severity might identify the degree of dysfunction occurring at each extreme of the bipolar continua.

### 3.3. Increasing efficiency of measures

Wider adoption of the dimensional PD is likely impeded by the length of the primary measures. This is a limitation of many personality measures. For example, the PID-5 (52, 126) has 220 items and the CAT-PD has 216 items. Shorter measures have been developed, such as the 100-item PID-5 short-form (116), which has impressive psychometric equivalence to the parent measure. There are also much shorter variations such as the 25-item PID-5 brief form (52). Nonetheless, measurement length reduces researcher enthusiasm for comprehensive PD trait assessment, which has generated shorter measures [such as the Five-Factor Form; (120)]. This concern appears more strongly situated with PD traits than severity, as there are a range of efficient severity measures in development [e.g., PDS-ICD-11; (84)]. Given the increase in research efforts toward intensive longitudinal designs and the pressure for shorter measures, efficient measurement is essential to reduce participant burden and to increase the feasibility of research projects.

Shortening scales has several psychometric considerations. Firstly, this process can come at a cost to measurement precision, introducing unnecessary error variance into an already complex measurement space. Item selection also needs to balance content coverage with item performance. Selecting items based only on their performance (such as high factor loadings or correlations) can come at the cost of measurement breadth because these items do not always cover all content domains. This limitation is regularly overcome by sacrificing facet scores in favor of trait level estimations [e.g., IPIP-NEO; (127); PID-5 brief form] or moving to single-item or two-item measures. This is not to say that short forms cannot provide psychometrically robust estimation, but it is substantially more difficult to achieve that with broader constructs.

Computerized Adaptive Testing (CAT) appears to be a promising solution. This is an adaptive testing process where items are iteratively administered to participants based on their previous scores (128, 129). With the increase in computing power and online or phone-based surveys, linear approaches (completing all items in a pre-set order) could be considered antiquated. CAT is based in Item Response Theory (IRT), using pre-calibrated item sets to tailor item administered to each participant individually. As a result, participants are provided only the items that provide meaningful information about their trait, reducing administration times in previous personality trait studies as much as 60% (130, 131). Further efficiencies can be found through multi-dimensional and bifactor CAT models (132), particularly when there are correlated traits. Further, most CAT models are simply adapted from linear tests. This undermines their potential reliability and accuracy because standard item content focuses on full trait coverage. Instead, CAT item pools could comprise items designed to have “surgical precision”, with content solely focused on narrow trait ranges (e.g., only differentiating distressed participants from those who are highly distressed). In doing so, CAT will only display these items to participants in the applicable narrow band on the trait, having the potential for more accurate estimates than their linear ancestors.

Despite initial enthusiasm, interest in this measurement approach appears to have faded, with little use of CAT versions of the SNAP (131) or the Computerized Adaptive Assessment of Personality Disorder (79). We encourage researchers entertain the use of CAT because of its substantial benefits and because of the proliferation of easy-to-use open-source tools [see (133, 134)]. The benefits can include reducing assessment burden with intensive longitudinal designs (101) and within longer survey batteries.

## 4. Clinical utility of dimensional models: Challenges and opportunities

At the time of the publication of the DSM-5, skepticism regarding the utility of dimensional models were high. There were several epicenters for this concern: (1) that categorical or hybrid was favored more than trait-based models by clinicians (135, 136), (2) that the removal of many important diagnoses was premature and unjustified (137), and (3) that dimensional models do not capture the full range of diagnoses adequately and are overly complex (138). This complexity is a serious issue for clinicians, as complexity combined with a learning hurdle will reduce the likelihood of routine clinical adoption (139). For example, Bernstein et al. (58) found that expert members of two international PD associations largely felt that the current DSM-IV categorical model should be replaced and supported a dimensional perspective. Most respondents, however, preferred a mixed classification system, comprising dimensional and categories [similar findings by Morey and Hopwood (140)]. In line with these results, the AMPD included the hybrid system as a stepping-stone between the two approaches (22, 141) until research for the dimensional perspective's clinical utility was sufficiently convincing.

Since inception of the dimensional perspective, an impressive body of research has accumulated. This is not an understatement. In a recent review, Bach and Tracy (24) identified an astonishing 1,281 articles on the clinical utility of the AMPD. In contrast to earlier studies, they concluded that dimensional approaches were seen as more useful than categories for many aspects of clinical utility. For example, they are particularly useful in treatment formulation, monitoring, and communicating with both professionals and families (60, 140). Largely, severity ratings (e.g., Criteria A or ICD-11 severity) act as a benchmark for severity and impairment to allocate public health resources, and to warrant levels of intervention (e.g., medication and in-patient treatment). The trait profile would act to guide treatment plans and communication.

Although the benefits largely outweigh the costs of moving toward dimensionality, it is difficult to abandon categories due to their allure of simplicity. Consumers and health professionals tend to prefer an uncomplicated and straightforward lexicon for communicating and understanding mental-health issues. An array of trait levels will likely not meet this need. Challenging these concerns, a recent study (142) asked 163 mental health professionals (e.g., nurses, doctors, and psychologists) to apply ICD-10 and ICD-11 PD frameworks to one of their existing clients. When compared to the ICD-10 classification system, the ICD-11 dimensional framework was rated as marginally more useful for treatment planning, ease of use, and communication with patients and with other professionals. The implementation of a new system, despite its positive reception, will lead to a disconnection between current and previous research on recommendations, treatments, and policy (34). However, this could be seen as an opportunity for validated research and treatments to be incorporated into an evidence-based approach, while disregarding non-reproducible findings and unsupported theory.

Work on the direct application of these models needs further research and trial, despite evidence for their endorsement by clinicians (24, 143, 144). We will briefly outline several areas of further inquiry to smooth the transition in the next DSM iteration. We see much of this research on the near horizon, catalyzed by the ICD-11 installation of a dimensional system that will have to be implemented for all WHO members. Therefore, dimensional approaches will be used for national statistics, treatment allocation, and for billing practices.

### 4.1. Formulating personality disorders using traits

Both the DSM-5 AMPD, ICD-11, and HiTOP frameworks offer a broad-building blocks for the foundation of the new approach to psychopathology. Yet, it is unclear how these building blocks should be organized into a coherent conceptual understanding of an individual's PD. For example, many of the traits and their facets can be both underlying temperamental and more variable defense or coping mechanisms (145, 146). Intimacy avoidance (PID-5 facet) could act as a defense mechanism against rejection or assault, or rigid perfectionism might develop to compensate for perceived inadequacy or due to overvaluing success. In this way, traits (and facets) are likely the result of varying mixtures of underlying temperaments and how that person has learnt to meet their needs (146–148).

The dual developmental process obscures the genesis of that trait, and the mechanism of life dysfunction (145). Neurological or neurochemical temperaments may need different treatment methodology to defense mechanisms. Interestingly, most current psychotherapies do not aim to change traits, and instead focus on how intrapersonal and interpersonal problems are being generated and maintained. A notable example is the modularized approach within dialectic behavioral therapy [DBT; (149)], which address specific issues that arise within borderline personality disorder (such as emotional regulation, distress tolerance, and interpersonal skills). Several studies have suggested that although traits might remain relatively stable, distress and impairment can vary substantially [e.g., (31)].

Intrapersonal and interpersonal problems naturally provide primary treatment targets given their direct linage with distress. Traits and PD severity, however, are not clearly demarked in current assessment approaches because distress is also imbedded within the trait items themselves (e.g., “I can't stand being left alone, even for a few hours”). This is also evident in the limited incremental validity generated when assessing both severity and traits, sparking recent debates about the utility of both approaches [see (18)]. The integration of distress within maladaptive trait models does suggest that treatment centered around these traits are likely beneficial. This would, in essence, reduce an individual's maladaptive trait back to their underlying FFM dispositions and efforts to link treatment approaches to specific traits have already begun (150, 151).

PDs treatment might benefit from a reconceptualization as interpersonal disorders (152–154). Proponents of this change highlight that most PDs are inherently interpersonal, either directly through interpersonal behavior (e.g., antisociality or avoidant) or indirectly (affective dysregulation due to perceived abandonment). Further, aspects of PD that are not inherently interpersonal are already featured under other diagnostic labels (e.g., Schizotypal PD). Redefining personality-related distress as interpersonal in nature enables direct mapping of treatments to issues. For example, clinical treatment can directly target issues with a person's capacity to managing social processes (understanding the situation and engaging in adaptive processes) or self-processes (understanding themselves and regulating motivations and affect) (154). This approach has substantial practical benefits, in addition to the potential for reducing stigma associated with labeling a person as inherently disordered (as discussed in Section 5 below).

In terms of implementation, the hierarchical nature of these models allows for a graduated approach to assessment based situational demands (150, 155). For example, in time-limited situations such as acute settings, PD severity might be all that is needed in addition to risk assessment. This would justify health-care intensity (e.g., inpatient, outpatient) and immediacy. If the goal is then to identify the nature of the patient's issues, trait level analysis or HiTOP syndromes or components could be used. As raised earlier, this should be guided by multi-method assessments to account for weakness within a single approach. Trait-level assessment would also guide multidisciplinary involvement and higher-level treatment planning, and identify interpersonal tendencies that might interfere with or aid therapy. A lower-level or facet understanding can be used to generate a more complete formulation or understanding of the person, their issues, and viable evidence-based interventions for specific issues. Treatment can then focus on specific traits or facets rather than being linked to categorical disorders. This is similar to cross-cutting interventions that currently exist, such as the transdiagnostic unified protocol (156). This stepped approach is inherently adaptive and guides treatment toward the nature of distress instead of on categorical labels.

### 4.2. Training and funding

Several studies have demonstrated the positive reception and trainability of AMPD. For example, Morey (157) found that college students ratings, without any exposure or training, of a target acquaintance on the DSM's Criterion A (LPFS) were internally consistent, and reliably differentiated between levels of severity. Zimmermann et al. (158) asked clinically inexperienced and untrained students to rate the personality functioning of video-taped inpatients on a derivation of the LPFS. The results suggested strong interrater correlations and convergence with expert clinician ratings. These results suggest that little experience or training is required, and that applying Criterion A to patients is relatively straight forward. We encourage similar work that involves testing the learning hurdles and complexity of applying reliable trait estimates (Criterion B).

Despite this ease of application, categorical models are still widely taught and applied in clinical and teaching spheres. Broader acceptance in training programs would increase familiarity with the model and provide naturalistic studies on the adoption and utility of this model from new clinicians. Instead, current research is limited by brief introductions to the models and vignettes, and focusing on trainees and not seasoned clinicians (158). These programs would also provide the capacity for broad studies into the acceptance and refinement of the model by clinicians and patients. Instead of small-scale studies of clinician perspectives on utility, these studies could compare patient communication and outcomes across treatment sites. Feedback tools would then be developed to further the positive reception of the individualized communication and feedback from FFM based assessments (159). Research-focused institutions such as university clinics and teaching hospitals appear to be an ideal location for this work.

Adopting dimensional PD approaches in clinical training programs could be stimulated by developing and disseminating treatment approaches. As previously discussed, treatment approaches and guidelines are in rapid and active development [e.g., (150, 160, 161)], which have provided the foundation for ongoing research into their efficacy (24). Nonetheless, convincing clinicians and developers of training programs to use these approaches requires a strong demonstration of treatment efficacy above and beyond categorical approaches. Despite studies into the perceived usefulness of these approaches, almost no work has actually demonstrated increased treatment efficacy. This undertaking would require randomized controlled trials across multiple sites (162), which would investigate changes in personality functioning and impairment through trait and distress informed treatments (161).

Targeting idiosyncratic trait profiles or domains of impairment for personalized care programs risks difficulties with standardization. One potential solution is to conduct trials of modularized treatments for specific trait and impairment combinations. Strong candidates for these modular treatments have been proposed that integrate existing evidence-based approaches with dimensional nosology [see (163)]. Modularized treatments that prove effective can be integrated into standard treatment recommendations, and ongoing research can focus on adding case complexity (such as multiple elevated traits and environmental pressures). Regardless, this remains a substantial remaining barrier to broader dimensional adoption in health-care systems.

The removal of clear categorical diagnoses in the DSM and ICD has implications for funding, potentially making research on PD severity less attractive to funders. Nonetheless, the DSM and ICD PD severity codes (mild, moderate, severe) provide an initial method for indexing impairment and prognosis, serving as the foundation for public mental health support through a graduated support model. This change in funding allocation supports research on PD severity being a better indicator of impairment than categorical diagnoses (36). By assigning qualitative labels to severity, dimensional PD diagnoses can be operationalized categorically in the same manner as mild, moderate, and severe depressive episodes. Similarly, distinguishing between elevated or normal range trait specifiers facilitates a similar categorical distinction to guide treatment and funding.

Nevertheless, a conceptual and empirical problem arises by assigning categorical groupings to an inherently dimensional continuum. Initial work has used IRT to estimate potential elevation-based thresholds for severity, such as the PDS-ICD-11 (82). An important avenue of future research is to match these thresholds to clinician-based ratings and real-world impairment/empirically derived severity estimates, as well as to link dimensional PD severity to prognosis, support requirements, and treatment responsiveness (161, 164). It is also important to identify a protocol for managing individuals on the border of two trait or severity categories (e.g., moderate – severe). Increased impairment necessitates increased resources, but more research is required to understand the degree and form of this support.

In the short term, we will likely see a “cross-walk” approach that translates severity and trait dimensions into specific DSM/ICD categorical labels (or DSM hybrid types) for funding in many countries. This is simply because of the integration of categorical diagnoses throughout the mental health care system, and its familiarity with clinicians and patients. Such cross-walk approaches already exist, such as for the broader Hierarchical Taxonomy of Psychopathology (HiTOP) framework (155). In contrast to the DSM, WHO member countries are required to use the ICD-11 severity codes for legal purposes, insurance, and national health statistics. With this broader adoption of dimensional frameworks into clinical practice, research can investigate the direct relationship between public mental health usage, severity, and treatment efficacy. This will allow for broader mapping of the most efficient use of health-care resources for effective outcomes and client support.

## 5. Inclusivity of dimensional models: Challenges and opportunities

### 5.1. Inclusivity through dimensional models' universal and cross-cultural applicability

The AMPD Criterion B and the ICD-11 trait domain specifiers have their theoretical basis in the Five-Factor Model (26), a dominant personality trait model in psychology. Although widely-accepted and touted as a universal model of personality, with the five basic and biological dispositional personality traits (165, 166), its universality and cross-cultural applicability is far from certain. Many researchers have questioned its claims for universality and its imposition of a particular structure identified originally in the English language and in North American samples onto the rest of the world [e.g., (167–169)]. Indeed, numerous studies [e.g., (44–46, 170, 171) have failed to replicate the five-factor structure—especially in non-Western societies and particularly in those that are culturally distant from the West, such as the Tsimane foragers of Bolivia (45) and the Ache of eastern Paraguay (170). Nonetheless, there are also convincing arguments that this model even does not appropriately explain personality variation in Western societies, with, for example, some proposing a six-factor model as more precise and comprehensive model of personality (172), and others proposing a three-factor model (44).

Similarly to the work on the Five-Factor Model, much of the research into the dysfunctional trait models, which we have reviewed, has focused on North American samples. Nonetheless, researchers have demonstrated that the AMPD trait model and its most widely-used measure, the PID-5, appear useful in other countries and languages. For example, the PID-5 and/or its shorter forms have been successfully used in many countries and languages, such as Poland (173), Spain (174), Sweden (175), France (176), Germany (177), Italy (178), Iran (179), Czech Republic (180), Brazil (181), Russia (182), and three Arabic-speaking countries (183). Similarly, a quantitative review of the PID-5 in US and non-US (almost exclusively Western European) samples showed evidence for a five-factor structure of the PID-5 scales in all samples (54). This replicability is no doubt impressive, but like countless other measures in psychology and psychiatry, the measure itself was developed in the US, in samples with predominantly US White participants (52), and then exported to other groups and cultures. Similarly, the new PDS-ICD-11 (82) and preliminary ICD-11 scales (87) have been developed in western samples. This kind of research into personality psychology has been frequently criticized by cross-cultural psychologists (167–169), as it fails to take into account the conceptualization of personality and unique social contexts of cultures under investigation. That a particular measure performs well in other cultures does not mean that the underlying conceptualization itself is valid but only that the measure may be administered successfully across cultures (184).

Although both the DSM-5 and ICD-11 claim that the role of culture is central to the assessment of PDs, researchers have paid much less attention to this role. We agree with Choudhary and Gupta's (185) assessment that: “Despite the importance of culture, much of the theory and research about PD have severely underestimated or even ignored the influence of social organization and culture” (p. 3). As with all personality research (169), the dominant approach to investigating the AMPD is largely based on the imposed-etic approach, where conceptualizations and measures developed in one culture and language are being exported to other cultures and languages. At times, exporting the AMPD has been shown explicitly not to work that well. For example, there have been questions about the extent to which the PID-5 works in ethnic minority groups in North America, with a recent study showing strong performance of the measure in White American samples, but not in Black American samples, in which the five-factor structure could not be extracted (186). Similarly, a recent study employing the PID-5 brief form in China (187) found stronger support for a six-factor model, which the authors argued was more in line with Chinese conceptualizations of personality, where the factor of interpersonal relationships plays a more unique and significant role than in Western countries. To our knowledge, there has been no research into the PID-5 in non-industrial societies, and it is an open question whether the measure's five-factor structure would apply to these societies given that it may not work well even in an ethnic minority group in the US or in China, and given that the Five-Factor Model has little support in non-industrial societies.

Unfortunately, due to many influences, including institutional and individual, ethnocentric approaches affect the study of psychology in general and at all levels, such as the topics of study, theoretical frameworks, and the choice of methods, including participants, materials, and procedures (188, 189), and the study of personality in particular (184, 190, 191). As a result, we know much more about personality in Western countries than in non-Western countries. This state of affairs is unfortunate but with the DSM-5 and ICD-11 enforcing cultural aspects in the assessment of PD, it is of extreme importance for researchers to pay significantly more attention to the role of culture. This kind of research requires an international endeavor and cross-cultural research, where an equal voice is given to experts and researchers from different cultural traditions in formulating culture-specific, and agreeing on culture-general (i.e., universal), conceptualization, theorizing, and measurement of personality, personality pathology, and impairment in personality functioning relative to what is normative in a particular cultural context [cf. (192)]. Cross-cultural development would further evaluate theory that maladaptive traits describe individual differences in the resting state and reactivity of universal biological systems, such as the flight-or-fight mechanism underlying neuroticism (41). Nonetheless, only with employing this kind of research we can begin transcending ethnocentric barriers and limited cross-cultural generalizability of the AMPD, and consequently may gather further international support for the model.

### 5.2. Inclusivity through stigma reduction

A gap in the literature, with virtually no published research, is whether the dimensional PD approaches would make people with PD experience more inclusive attitudes and less stigma. Stigma comprises three components: stereotypes, prejudice, and discrimination (193), with possibly prejudice being the core of stigma due to its negative evaluative component, which drives discrimination. Although mental health professionals, compared to the general population, tend to engage in less prejudice toward people with mental disorders overall and toward people with depression and schizophrenia in particular (194), there has been much less work on stigma and prejudice in relation to people with PD among both mental health professionals and in the general population. Studies have shown that people with PD, especially with borderline PD (but also people with narcissistic, antisocial, and paranoid PD), are likely to be seen as “difficult patients” (195–197), which in turn makes clinicians less likely to want to work with them and this can result in poorer provision of care. In addition, people with PD, especially with borderline PD, are often more likely to experience stigma than people with many other serious mental disorders (198).

Although we know little about whether the dimensional PD approaches would reduce stigma, a recent systematic review shows that continuum beliefs about mental disorders tend to reduce stigma compared to categorical beliefs (199). Regrettably, as revealed by Peter and colleagues, the published research investigating this question has largely focused on people with depression and schizophrenia, with only few studies also looking at people with other mental disorders (alcoholism, attention deficit/hyperactivity disorder, obsessive-compulsive disorder, and dementia), and with no published study investigating PD. It can nevertheless be theoretically expected that even in relation to having continuum beliefs about PD, prejudice and stigma may decrease among mental health professionals, people with PD themselves, and the general population. This is in line with the social identity approach (200–202), which assumes that people constantly create psychological groups. When people identify with particular groups, they depersonalize and their self-interest is changed into group self-interest. This process combines with the need for positive group distinctiveness, leading people to prefer ingroups to outgroups. Accordingly, the categorical model demarcates who the ingroup (those who are outside the category of PD) and outgroup (those who are inside the category of PD) are, leading to more stigma against the outgroup. The dimensional approach would theoretically lead to perceiving people with PD as like “us” and not outgroups, as all people share these traits and this in turn may decrease stigma and prejudice. Similarly, people with PD may be less likely to identify with PD if they feel that all people share these traits to a certain extent. Research shows that identification with a disorder can lead to integrating that disorder's identity and therefore poorer wellbeing (203). Accordingly, the dimensional PD approaches may lead to less identification with a disorder in people with PD.

Beneficial identities could also be fostered by modifying nomenclature (204). Given the strong association between PD and interpersonal difficulties, the term “Interpersonal Disorders” might be more appropriate than PD (152, 153). The proposed change shifts the source of the dysfunction from the individual to their difficulty. In doing so, we discontinue the problematic practice of labeling the individuals themselves as the issue, reducing the likelihood that they will internalize unhelpful social identities or stigma. An Interpersonal Disorders label also externalizes the issue, providing a clear treatment target for the client and the treating clinician. This is an interesting proposal, requiring ongoing research into where symptoms not directly related to interpersonal distress (e.g., impulsivity and schizotypal) fit within broader psychopathological frameworks (such as HiTOP).

Nonetheless, questions remain as to whether the dimensional perspectives would indeed reduce prejudice and stigma for individuals with PD. It is plausible that people can still differentiate between those who are lower on particular PD dimensions (us/ingroups) from those who are higher on these dimensions (them/outgroups), and this in turn may increase stigma against outgroups. Also, research shows that people with PD tend to experience higher stigma even before being diagnosed with a PD, possibly because they are likely to internalize negative feedback from others on their behavior and emotional reactions (198). Given different findings and theoretical expectations, future research should carefully and systematically investigate if dimensional perspectives would indeed lead to less stigma and prejudice in mental health professionals but also in people with PD and the general population. If research shows that the dimensional perspectives indeed lead to less stigma and prejudice as theoretically expected, we can expect that it may contribute to the wider adoption of the model.

## 6. Conclusion

Overall, the dimensional model offers an evidence-based framework that provides the potential for effective personalized treatment through unifying and not dividing individuals. The future of dimensional approaches appears optimistic, with the growing evidence alleviating many of the concerns raised before the DSM-5 release. We highlighted three areas for ongoing development, that is, measurement, clinical utility, and inclusivity. We specifically advocated for diversifying measurement, testing treatment efficacy and health system linkages, developing cross-cultural models driven by both Western and non-Western cultures, and investigating whether dimensional perspectives may potentially reduce stigma leading to positive societal outcomes. We hope that this will direct researchers toward furthering these goals and transcending barriers to wider adoption. We further encourage these research efforts to be consumer-led or consumer-informed, reducing the divide between research and practice.

## Author contributions

CM: structure, design, writing, and editing. BB: design, writing, and editing. All authors contributed to the article and approved the submitted version.
